# Generation and selection of anti-flagellin monoclonal antibodies useful for serotyping *Salmonella enterica*

**DOI:** 10.1186/2193-1801-2-640

**Published:** 2013-11-28

**Authors:** Yanina Hiriart, Maria Serradell, Araci Martínez, Sofia Sampaolesi, Dolores Gonzalez Maciel, Jose Alejandro Chabalgoity, Lucía Yim, Gabriela Algorta, Martin Rumbo

**Affiliations:** Laboratorio de Investigaciones del Sistema Inmune (LISIN), Facultad de Ciencias Exactas, Universidad Nacional de La Plata, La Plata, CP1900 Argentina; Departamento de Ciencias Biológicas, Cátedra de Microbiología, Facultad de Ciencias Exactas, Universidad Nacional de La Plata, La Plata, Argentina; Departamento de Bacteriología y Virología, Instituto de Higiene, Centro Nacional de Salmonella, Facultad de Medicina, Universidad de la República, Montevideo, Uruguay; Departamento de Desarrollo Biotecnológico, Instituto de Higiene, Facultad de Medicina, Universidad de la República, Montevideo, Uruguay

**Keywords:** Salmonella, Serotyping, Flagellin, Monoclonal antibodies

## Abstract

In developing countries, bacterial acute gastroenteritis continues to be an important cause of morbidity and mortality among young children. Salmonellosis constitutes a major cause of infectious enteritis worldwide, most of them associated to the consumption of contaminated food products. Traditionally, *Salmonella* has been classified in serovars based on varieties of O and H surface antigens. In the present work we generated and characterized a panel of anti-flagellin monoclonal antibodies (MAbs) in order to select antibodies useful for detecting the H surface antigen. Four different MAbs were obtained by somatic hybridization of splenocytes. We found two MAbs that recognised regions of flagellin conserved among different *Salmonella* serovars. Other two MAbs recognised structures restricted to *Salmonella enterica* sv. Typhimurium, being one of them suitable for agglutination tests. Using a diverse panel of *S. enterica* serovars with different H antigen varieties we confirmed that this MAb agglutinates specifically *S.* Typhimurium (antigenic formula: 4,12:i:1,2) or other serovars expressing flagellar factor i. In conclusion, we generated a valuable immunochemical tool to be used in simple assays for serotyping of epidemiologically relevant strains. The capacity to characterize specific strains and determine the primary sources of *Salmonella* contamination generate valuable information of the epidemiology of this microorganism, contributing to the improvement of public health.

## Introduction

*Salmonella enterica* is an important etiological agent of gastrointestinal infections worldwide that may also evolve to invasive disease. Each year more than 90.000.000 enteritis and about 150.000 deaths are caused by this microorganism (Majowicz et al. 2010). It has been estimated that more than 95% of human cases of salmonellosis are originated by the consumption of contaminated food products (Mead et al. 1999), most of them associated to ingestion of poultry, beef, pork, eggs, milk, seafood, and fresh produce (Foley et al. 2007). Understanding how *Salmonella* disseminates through the food chain is critical to determinate how food processing procedures contribute to infection. To this aim, the capacity to characterize specific strains and determine the primary sources of *Salmonella* contamination generate valuable information of the epidemiology of this microorganism, being a basic tool to improve public health.

Traditionally, *Salmonella* has been classified in serovars using defined procedures of agglutination, based on the different antigenic varieties of O and H surface antigens that correspond to LPS and flagellin molecules (Brenner et al. 2000). Serovars are designated using the Kauffmann–White-Le Minor classification scheme, usually employed by public health organizations to serotype *Salmonella* (Guibourdenche et al. 2011). The use of genetic information to group isolates has determined the separation of the *Salmonella* genus in two different species, *Salmonella enterica* and *Salmonella bongori*, with more than 99% of the serovars belonging to *S. enterica* specie (Foley et al. 2007). Although more than 2500 serovars of *Salmonella* have been described, more than 50% of the human infections reported worldwide in the last few years were caused by the two most prevalent serovars *S. enterica* serovar Enteritidis and *S. enterica* serovar Typhimurium (*S*. Enteritidis and *S*. Typhimurium respectively) (Hendriksen et al. 2011).

Flagella confer motility to *Salmonella* and have been employed as one of the main antigenic determinants used for serotyping *Salmonella* isolates. The external body of the flagellum is mainly constituted by an homopolymer of flagellin, organized in an helicoidal distribution, presenting an exposed central domain that is highly variable among the different serovars and conserved N and C-terminal regions that are responsible for the polymerization of flagellin to form the flagellum (Ramos et al. 2004). The variability of the central domain of flagellin is reflected in the high number of serovars described for the *Salmonella* genus. Traditionally, serotyping was conducted using polyclonal antisera that were generated by inoculation with the desired strain and adsorbed with a set of related strains to increase the specificity of the resulting antiserum (Gray et al. 1974). In the case of H antigen, preparation of specific antiserum was facilitated by the use of purified flagellin for immunization (Ibrahim et al. 1985a), although the presence of conserved regions in the molecule among different serovars originated possible cross-reactions depending on the procedure used for antiserum generation (Ibrahim et al. 1985b). Upon the widespread use of techniques for monoclonal antibody (MAb) generation, several MAbs for strain serotyping have been described (Sojka et al. 2001; de Vries et al. 1998; Rementeria et al. 2009; Iankov et al. 2002). The availability of strain-specific MAbs for serotyping provides many advantages, such as using a molecularly defined homogeneous reagent that can be produced in high scale and basically without restrictions of quantity (Nelson et al. 2000).

In the recent years, several genotype based molecular methods have been developed in order to identify *Salmonella* strains (reviewed in Foley et al. 2007). However, the traditional serotyping methodologies are still widely used, especially in low resources developing countries, consequently, the development of tools to provide accurate microbial identification to these settings remains of interest. The aim of the present work was to generate and characterize a panel of anti-flagellin MAbs in order to select antibodies useful for diagnostic purposes. We have tested specificity of these antibodies by different immunochemical techniques and also detected a MAb that is suitable for agglutination of the epidemiologically relevant *S.* Typhimurium (antigenic formula: 4,12:i:1,2) or other serovars expressing flagellar factor i, that would be useful for serotyping purposes.

## Materials and methods

### Bacterial strains, media, and growth conditions

Bacterial strains used in this study are listed in Table 1. The strains were grown at 37°C in tripticase soy agar (TSA) plates or Sven-Gard (0,7% agar in nutrient broth) plates for evaluation of somatic or flagellar antigens respectively, using agglutination tests. All *Salmonella* isolates were confirmed biochemically and serologically at the National *Salmonella* Centre (NSC, Instituto de Higiene, Universidad de la República, Uruguay). For flagellin obtention for immunizations, a *S.* Typhimurium mutant strain SIN22 [fljB5001_MudJ in ATCC14028, M], carrying a deleted *fljB* gene (phase 2 flagellin, that defines the antigens 1,2), constructed from *S*. Typhimurium ATCC14028 (American Type Culture Collection, Manassas, VA) was employed (Didierlaurent et al. 2004). This mutant contains the ampicillin resistant plasmid pRP2 carrying the *fliC* coding sequence for *S*. Typhimurium phase 1 flagellin (that defines the antigen i), under constitutive promoter.

**Table 1:** ***Salmonella*** **strains used in this study**

Strain	Description (antigenic formula)	Source and/or reference
*S*. Typhimurium	NSC Reference strain (4,12:i:1,2)	Institute Pasteur, Paris^*^
*S.* Frankfurt	NSC Reference strain (16:i:e,n,z15)	Institute Pasteur, Paris
*S.* Infantis	NSC Reference strain (6,7:r:1,5)	Institute Pasteur, Paris
*S.* Makiso	NSC Reference strain (6,7:1,z13,z28:z6)	Institute Pasteur, Paris
*S.* Newport	NSC Reference strain (6,8:e,h:1,2)	Institute Pasteur, Paris
*S*. Paratyphi B	NSC Reference strain (4,12:b:1,2)	Institute Pasteur, Paris
*S*. Enteritidis	NSC Reference strain (9,12:g,m:-)	Institute Pasteur, Paris
*S.* Derby	NSC Reference strain (4,12:f,g:-)	Institute Pasteur, Paris
*S.* Bergen	NSC Reference strain (47: i:e,n,z15)	Institute Pasteur, Paris
*S.* Diarizonae serovar IIIb	NSC Reference strain (48:i: z35,z57)	Institute Pasteur, Paris
*S.* Typhimurium SL1344	Reference strain (NCTC 13347) (4,12:i:1,2)	National Collection of Type Cultures (NCTC), England
*S*. Enteritidis CIDCA 101	(9,12:g,m:-)	CIDCA, CCT La Plata, CONICET^**^
*S.* Bredeney	(4,12:l,v:1,7)	Instituto Biológico Dr. Tomás Perón, La Plata^***^
*S*. Saintpaul	(4,[5],12:e,h:1,2)	Instituto Biológico Dr. Tomás Perón, La Plata
*S*. Newport	(6,8:e,h:1,2)	Instituto Biológico Dr. Tomás Perón, La Plata
*S*. Anatum	(3,10:e,h:1,6)	Instituto Biológico Dr. Tomás Perón, La Plata
*S*. Typhimurium SIN 22	(4,12:i:1,2)	Didierlaurent et al. [20]
*S*. Typhimurium SIN 41	(4,12:i:1,2)	Didierlaurent et al. [20]

^*^strains obtained from the Centre International de *Salmonella,* Institute Pasteur, Paris, (CIS) belong to the NSC collection.

^**^strain belonging to the Centro de Investigación y Desarrollo en Criotecnología de Alimentos (CIDCA) collection, La Plata, Argentina.

^***^strains isolated from different food sources in the Departamento Laboratorio Microbiológico, Dirección de Laboratorio y Control, Instituto Biológico Dr. Tomás Perón, La Plata, Argentina.

As isogenic control, the mutant *S.* Typhimurium strain SIN41[fliC5050::MudJ fljB5001::Mud-Cam], knock-out for *fliC* and *fljB* genes, constructed from *S*. Typhimurium ATCC14028 was used.

### Flagellin purification

Flagellin was prepared from different microbial strains as previously described (Hiriart et al. 2012; Nempont et al. 2008). Briefly, bacteria were grown for 16 h at 37°C with agitation in LB media. Flagella were sheared from surface, pelleted by ultracentrifugation, and heat treated to release flagellin monomers. Flagellin was stored at -80°C until used. A recombinant phase I flagellin from *S. enterica* serovar Typhimurium with a deletion in aa 200 to 369 (FliC C131) (Malapaka et al. 2007) was produced in *E. coli* and purified by ion exchange chromatography from urea 6 M refolded inclusion bodies obtained after overnight bacterial culture. The coding sequence for this flagellin was a kind gift of Dr. Jean Claude Sirard (Institut Pasteur Lille, France).

### Generation of MAbs

Mice were provided by the School of Veterinary Sciences of the National University of La Plata animal facility. All the experiments were performed according to the guidelines set by the National Institutes of Health (NIH publication Vol 25, N° 28 revised 1996) and were approved by the institutional commission of animal welfare (CICUAL). Monoclonal antibodies were derived by somatic cell hybridization as described by Galfré and Milstein (Galfre and Milstein 1981) using polyethylene glycol (MW 3350) as fusogenic agent. Briefly, BALB/c mice were immunized by intraperitoneal injections (separated every 25 days) with 1 μg of flagellin from *S.* Typhimurium SIN 22 without adjuvant (Didierlaurent et al. 2004). NS0 myeloma cells were fused with spleen cells from immunized mice and the resulting hybridomas were cloned by limiting dilution. Specific reactivity was tested by indirect enzyme-linked immunosorbent assay (ELISA) with flagellin from *S.* Typhimurium SIN 22 as antigen. MAbs were purified by affinity chromatography in a protein A-Sepharose column (GE Healthcare, USA) as described (Sorell et al. 1998).

### Indirect ELISA

Polystyrene microtitre plates (Microlon, Greiner, Denmark) were coated with flagellin preparations from different microbial strains diluted in carbonate buffer (pH 9.0) at 0.1 μg per well. Incubation was carried out at 37°C for 1 h. Then wells were washed three times with PBS plus 0.05% (v/v) Tween 20 (PBS-T) and incubated with 3% non-fat dry milk dissolved in PBS at 37°C for 2 h. The plates were washed three times with PBS-T and then 100 μl of hybridoma supernatant culture medium or purified MAbs solution were applied to each well and incubated at 37°C for 2 h. Washing was repeated as described above, and then 100 μl of the peroxidase-labeled secondary antibody (horseradish peroxidase-conjugated goat anti-mouse antibody; Santa Cruz Biotechnology) diluted 1:2000 was added to each well. The plates were incubated at 37°C for 1 h. After another cycle of washing, the reaction was visualized by adding a solution containing 1 mg/ml o-phenylenediamine (Sigma) and 1 μl ml^-1^ 30% H_2_O_2_ (Merck) in 0.1 mol l^-1^ citrate-phosphate buffer, pH 5.0. The reaction was stopped by the addition of 2 mol l^-1^ sulphuric acid. Absorbance was determined at 492 nm in a microplate reader (Sirio, BioArs, Argentina).

### Western blot

Purified flagellin preparations were dissolved in sample buffer (2% SDS, 10% glycerol, 5% 2-mercaptoethanol and trace amounts of bromophenol blue dye in 62.5 mmol l^-1^ Tris–HCl, pH 6.8), heated for 5 minutes at 95°C and subjected to gel electrophoresis. After SDS-PAGE, proteins were electotransferred from the gel to nitrocellulose membranes (Micron Separation Inc. Westborough, MA) for 2 h and then blocked in PBS containing 0.05% Tween 20 and 5% non-fat milk for 1 h at 25°C. The membranes were immunoblotted with the indicated antibodies overnight at 4°C. After washing, bound antibodies were visualized with HRP-conjugated antibodies against mouse immunoglobulin G (IgG) by using the Enhanced Chemiluminesence (ECL) Western Blotting System (Amersham Biosciences, Germany) or by incubation with 4-chloronaphtol solution as described (Chirdo et al. 1999).

### Slide agglutination tests

For bacterial agglutination based on flagellar antigens, the strains were grown overnight at 37°C in Sven-Gard agar plates, containing 20 ul of pure hyperimmune serum specific to phase 1 or 2 flagellar antigens when required. The following day, a loop of bacteria far from the inoculating spot was taken and resuspended to homogeneity in 50 ul of saline solution (8.5% NaCl) on a glass slide. The bacterial suspension was mixed with 50 ul of specific hyperimmune serum or the purified MAbs serially diluted when necessary, and presence/absence of agglutination was determined visually. The protocol for agglutination based on somatic antigens was the same, but the strains were grown in TSA plates (containing 1,5% agar).

## Results

**Figure 1:**
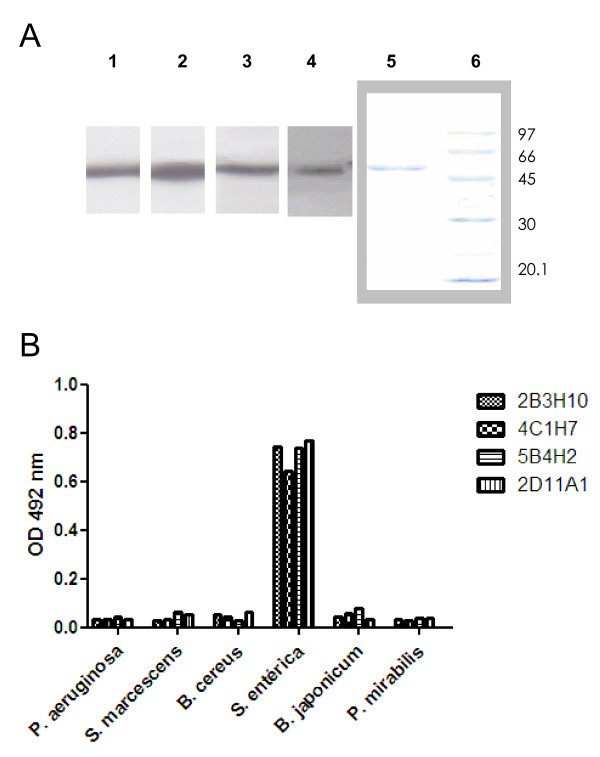
**The four MAbs recognize flagellin from** ***Salmonella enterica*** **and not from other species.** Panel **A**- Western-blot recognition of FliC from *S. enterica* serovar Typhimurium by the different MAbs (1- 2B3H10, 2- 4C1H7, 3- 5B4H2, 4- 2D11A1, 5- Coomasie blue staining FliC, 6- Coomasie blue staining MWM. Panel **B**- Characterization of the reactivity of the different MAbs against flagellin from different microbial species by indirect ELISA. Representative results of at least two independent experiments are shown.

Upon screening by indirect ELISA, four different MAbs were selected for analysis and characterization. Recognition of *S.* Typhimurium flagellin was confirmed by western blot (Figure 1A). Furthermore, purified flagellins from different bacterial species were used in indirect ELISA to determine the specificity of the recognition of the panel of MAbs under study. All MAbs tested showed high reactivity against flagellin from *Salmonella enterica*, whereas they showed very low reactivity against flagellin of other Gram- species such as *Serratia marcescens, Proteus mirabilis, Bradyrhizobium japonicum* or *Pseudomonas aeruginosa* or Gram + species such as *Bacillus cereus* (Figure 1B).

**Figure 2:**
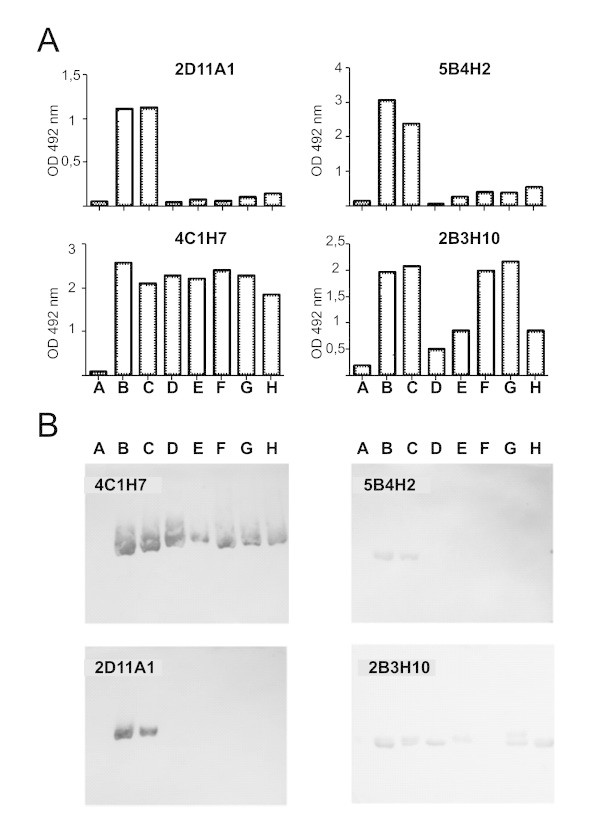
**Each MAb has a specific pattern of recognition of different** ***Salmonella*** **serovars.** Panel **A**. Recognition of flagellin from different strains by indirect ELISA. Panel **B**. Recognition of flagellin from different strains by Western-blot. In both panels, the same order of samples was used A- *S*. Typhimurium SIN 41 (negative control, flagellin deficient strain), B- *S*. Typhimurium SIN 22, C- *S*. Typhimurium SL1344, D- *S*. Enteritidis CIDCA101, E- *S*. Bredeney 655/05, F- *S*. Saintpaul 213/05, G- *S*. Newport 389/06, H- *S*. Anatum OS-A). Representative results of at least two independent experiments are shown.

Since flagellin varies among different *Salmonella* serovars, we wanted to determine the capacity of recognition of each MAb against flagellin from different serovars. We observed that each MAb has a specific pattern of recognition of different *Salmonella* strains (Figure 2). MAbs 4C1H7 and 2B3H10 have a broad capacity of recognition, being able to react against flagellins from different *Salmonella* strains, even from different antigen H serovars (such as *S.* Newport or *S*. Anatum, shown in lanes G and H respectively), whereas antibodies 5B4H2 and 2D11A1 are specific for Typhimurium, being this serovar the only recognised by these MAbs among all the variants tested (corresponding to positions B and C in Figure 2). The profile of reactivity was broadly concordant when indirect ELISA and western blot results were comparatively analyzed (Figures 2A and 2B). In the case of 2B3H10 broad reactivity was observed by ELISA and western-blot, however, there are some discrepancies between results obtained by both techniques, since flagellin from *S*. Enteritidis CIDCA101 (position D in Figure 2) is reactive against this MAb by western-blot but is weakly recognised by ELISA, whereas flagellin from *S*. Saintpaul 213/05 (position F in Figure 2) is reactive in the ELISA but weakly recognised in the western-blot. This data suggest that although 2B3H10 presents a broad capacity to recognition of different flagellin variants, it is susceptible to slight conformational changes that are particularly remarkable in these specific serotypes.

**Figure 3:**
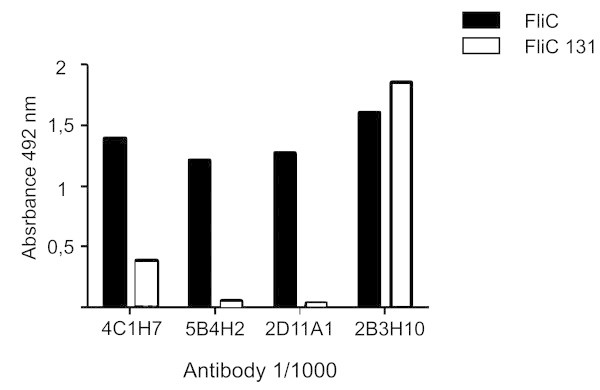
**Each MAb target different regions of flagellin.** Indirect ELISA using flagellin with a deleted antigenic exposed site (FliC C131) compared to wild type flagellin (FliC from S. Typhimurium SIN 22). Representative results of at least two independent experiments are shown.

We hypothesized that the broad reactivity shown by 4C1H7 and 2B3H10 may be due to its capacity to recognise flagellin regions conserved among different serovars. To test this hypothesis, we used a mutant flagellin that has a partial deletion in the D2 and D3 solvent-exposed domains, comprising the hypervariable region between positions 200 to 369 (Malapaka et al. 2007). This variant of flagellin contains the intact structure of domains D0 and D1, which normally participate in flagellum assembly and are highly conserved among different serovars. Using this mutated form of flagellin, by indirect ELISA, we could confirm that 4C1H7 and 2B3H10 recognise the conserved region of flagellin whereas 5B4H2 and 2D11A1 recognise the antigenic exposed domain (Figure 3).

As mentioned, agglutination tests are routinely used in diagnostic laboratories to serotype *Salmonella* isolates. Usually, incubation of a bacterial suspension with an adequate dilution of a specific antibody generates a network of antibodies linked to the surface of different bacteria, which are responsible for the agglutination reaction. Using a set of different *Salmonella* isolates, previously characterized using standard serological techniques at the NSC and routinely used as reference strains for serotype determinations, we further evaluated the agglutination capacity of MAbs 5B4H2, 2B3H10 and 2D11A1.

First, as positive controls, we used strains of *S. enterica* subsp. *enterica* serovars Typhimurium, Bergen, and Frankfurt, as well as a strain of *S. enterica* subsp. *diarizonae* serovar IIIb, all of which express the same phase 1 flagellar antigen (i), but in different contexts of somatic and phase 2 antigens (Table 2). The strains were grown in soft agar plates containing antiserum specific to the corresponding phase 2 flagellar antigen in order to repress expression of phase 2 flagella and thus promote expression of phase 1 flagella. In addition, we previously verified that flagellar antigen i was being expressed by agglutination tests using the specific hyperimmune serum routinely used at the NSC for serovar determinations. As shown in Table 2, it was clearly evidenced that 5B4H2 is the only MAb capable of bacterial agglutination based on i flagellar antigen, even when using dilutions of the MAb up to 1/1000.

**Table 2:** **Slide agglutination tests using** ***Salmonella*** **serovars expressing the i flagellar antigen**

***S***. Typhimurium						
MAb	Pure	1/10	1/100	1/800	1/1000	1/1200
5B4H2			+	+	+	+-
2B3H10	-		-			
2D11A1	-		-			
*S.* Frankfurt						
MAb	Pure	1/10	1/100	1/800	1/1000	1/1200
5B4H2			++	+	+-	-
2B3H10	-	-				
2D11A1	-	-				
*S*. Bergen						
MAb	Pure	1/10	1/100	1/800	1/1000	1/1200
5B4H2			+	+	+	+
2B3H10	-		-			
2D11A1	-		-			
*S.* Diarizonae serovar IIIb						
MAb	Pure	1/10	1/100	1/800	1/1000	1/1200
5B4H2			+	+	+	+
2B3H10	-	-				
2D11A1	-	-				

The MAbs were used pure or at the indicated dilutions. ++: intense positive agglutination, +: positive agglutination, -: negative agglutination, +-: uncertain.

We thus selected MAb 5B4H2 to evaluate its specificity of recognition by performing agglutination tests with strains of serovars of *S. enterica* subsp. *enterica* that express H antigens other than i and in different contexts of O antigens compared to Typhimurium (i.e. Infantis, Makiso, Newport and Paratyphi B). We selected Infantis and Makiso because serovars harboring flagellar factors r and z6 are those recommended by the Centre International of *Salmonella* (CIS) Institut Pasteur, Paris, as negative controls for testing specificity of i antiserum (Popoff 2001). Newport and Paratyphi B were selected because both express 1,2 as phase 2 flagella, similarly to Typhimurium. In addition, we tested strains of serovars epidemiologically relevant in our region (i.e. Enteritidis and Derby) (Betancor et al. 2010). We previously verified that serovars Newport, Infantis, Makiso, Paratyphi B, Derby and Enteritidis were expressing flagellar factors 1,2 / r / z6 / 1,2 / f,g and g,m respectively, by doing agglutination tests with the corresponding specific hyperimmune serums routinely used at the NSC for serovar determinations.

For the evaluation of the specificity of recognition, we used low dilutions of MAb, usually 1/10, and if some agglutination was observed, we increased dilution up to 1/1000. The results (Table 3) indicate that MAb 5B4H2 has good specificity for flagellar factor i, even if some minor cross reaction with flagellar factors 1,2 was observed at dilutions 1/20 or lower.

**Table 3:** **Slide agglutination tests using several** ***Salmonella*** **serovars expressing H antigens other than i and MAb 5B4H2**

Serovar	1/10	1/20	1/50	1/100	1/400	1/1000
*S*. Infantis	-					
*S*. Makiso	-					
*S*. Newport	++	+	-	-	-	-
*S*. Paratyphi B	+	+-	-	-		
*S*. Enteritidis	-					
*S*. Derby	-					
*S*. Enteritidis CIDCA 101	-					
*S.* Bredeney	-					
*S*. Saintpaul	-					
*S*. Newport	-					
*S*. Anatum	-					

The MAb was used at the indicated dilutions. ++: intense positive agglutination, +: positive agglutination, -: negative agglutination, +-: uncertain.

## Discussion

In the present work we generated a panel of anti-flagellin MAbs and selected one clone that is specific for serovars expressing flagellar factor i, among them the epidemiologically relevant *S*. Typhimurium. We also demonstrated that this MAb is suitable for agglutination tests, which may constitute a useful tool for the diagnostic laboratory.

Flagella are critical organs for *Salmonella*, conferring motility that is highly related with its capacity to spread among different hosts (Aizawa 1996). It has been shown that flagellin is a protective antigen against *Salmonella* infection, through T cell dependent mechanisms (Bobat et al. 2011). Although is a highly immunogenic protein, the diversity of flagellin variants among different *Salmonella* serovars may contribute to avoid the immune response elicited by one specific variant when a strain with a different flagellar variant confronts the host. As mentioned, this molecular diversity has been exploited to identify *Salmonella* strains and has been the basis of serovar classification (Brenner et al. 2000). On the other hand, the high immunogenicity of the exposed flagellin surface increases the chance of generation of MAbs recognising specific epitopes that are present in this exposed region. In our case, 2 different MAbs, among the panel of MAbs analyzed, recognised specific epitopes present in the central variable domain, as was shown using flagellin deletion variants (Malapaka et al. 2007) that were not recognised by these antibodies (Figure 3). The structural specificity of recognition of the MAbs generated was clearly specific for *Salmonella* flagellin, being unable to interact with flagellin of other species (Figure 1B). However, among the antibodies characterized, two MAbs that were directed against epitopes present in the variable domain were specific against *S.* Typhimurium flagellin using western-blot and indirect ELISA analysis; whereas the other MAbs that recognised structures in the conserved N or C-terminal region showed an overall broader specificity against flagellin from different *Salmonella* serovars (Figure 2), as it was expected.

The agglutination capacity of an antibody is dependent on structural parameters of epitope distribution in the surface of the bacteria and also on the accessibility of these epitopes, factors that are not easily characterized. In our case, of the different antibodies tested for agglutination, most of them were not able to generate a clear agglutination pattern, in spite of recognising by ELISA or western-blot the flagellin constitutive of the flagella of the tested strains. This could be explained by the fact that these antibodies recognise the conserved regions of flagellin, which are not exposed in the polymerised protein (Ramos et al. 2004). MAb 5B4H2 was highly efficient for generation of the agglutination pattern, which can be easily detected. MAb 5B4H2 was tested in a blinded format using 6 collection strains coming from either NCTC and two local sources (strains *S*. Typhimurium SL1344, *S*. Enteritidis CIDCA 101, *S*. Bredeney, *S*. Saintpaul, S. Newport M, and S. Anatum; Table 1), in a microbiology laboratory and a national reference center producing exactly concordant results and showing a clear specificity of recognition against i flagellar antigen.

Even if both MAbs 5B4H2 and 2D11A1 recognise the variable domain, as revealed by ELISA (Figure 3), predicting that both would be useful for bacterial agglutination, only the former was suitable for this application. It could be speculated that the exact epitope recognised by both MAbs is slightly different, and the one specific for 2D11A1 would be hidden in the assembled flagellar filament. In addition, the agglutination tests using serovars expressing flagellar factors other than i, indicated that even if the recognition was quite specific for i, some cross reaction occurs between MAb 5B4H2 and flagellar factor 1,2. However, at the dilution suitable for agglutination of strains expressing i factor (1/1000), this cross reaction is negligible, thus making this MAb a very useful tool for serotyping.

*S.* Typhimurium is one of the most important serovars of *Salmonella,* mainly because the involvement in episodes of foodborne disease. In a recent worldwide survey covering *Salmonella* infection in 37 countries participating in the World Health Organization Global Foodborne Infections Network (Hendriksen et al. 2011), it has been reported that *S.* Typhimurium has been responsible for around 17% of the infections of the reported *Salmonella* infections between 2001 and 2007, with a similar distribution along different continents with the exception of Oceania where it represented more than 50% of reported cases in the studied period. Along the period analyzed, a rise in incidence of this serovar in developing countries was observed.

Agglutination methods for *Salmonella* serotyping are still the main methodology employed for identification, especially in low resource laboratories in developing countries. The availability of specific MAbs that can be used in agglutination tests for serotyping, such as shown here, constitutes a valuable tool to identify isolates from clinical or field samples that will contribute to prevent the dissemination of salmonellosis.
